# Vertebral Artery Dissection and Takotsubo Syndrome Associated with Marijuana Use

**DOI:** 10.7759/cureus.4785

**Published:** 2019-05-30

**Authors:** Alejandro Sanchez-Nadales, Andrea Anampa-Guzmán

**Affiliations:** 1 Internal Medicine, Advocate Illinois Masonic Medical Center, Chicago, USA; 2 Internal Medicine, Main National University of San Marcos, Lima, PER

**Keywords:** broken heart syndrome, takotsubo cardiomyopathy, vertebral artery dissection

## Abstract

Cervical artery dissection is an intimal tear in a cervical artery with the development of intramural hematoma resulting in stenosis, occlusion, or aneurysmal dilation. Takotsubo syndrome (TTS) is characterized by acute heart failure with a distinctive regional left ventricular contraction profile. We presented the case of a 33-year-old male who visited the emergency department due to right-sided arm/leg weakness, slurred speech and transient loss of right eye vision. The patient was diagnosed with vertebral artery dissection and TTS complicated with community-acquired pneumonia. Only other case has been reported of TTS with bilateral vertebral artery dissection. TTS is a disease triggered by physical or emotional stressors. It can be confused with acute myocardial infarction and should be disoriented for the appropriate management. The prognosis is generally favorable.

## Introduction

Takotsubo syndrome (TTS), also called broken heart syndrome or stress-induced cardiomyopathy [1], is a cardiomyopathy that is characterized by acute heart failure with a unique regional left ventricular contraction profile, often accompanied by a significantly reduced left ventricular ejection fraction (LVEF). Although TTS has a presentation that is similar to an acute coronary syndrome, it is not caused by a coronary artery obstruction. As much as 90% of cases of TTS occur in patients that have recently experienced a severely traumatic event [2-3]. The exact cause is unknown, but the potential contributors include catecholamine excess and sympathetic nervous system hyperactivity.

The incidence of TTS is unclear since many cases may get misdiagnosed as an acute coronary syndrome. Ninety percent of TTS cases occur in postmenopausal women. In the United States, Takotsubo cardiomyopathy most commonly affects women over the age of 55 that have other risk factors for cardiovascular disease. Hyperlipidemia, smoking, alcohol use, anxiety, and stress are significantly associated with increased risk [4]. Less than 10% of patients are younger than 50 years [2]. TTS can be classified as primary or secondary depending upon the cause. In primary Takotsubo syndrome, patients primarily present with acute cardiac symptoms, of which the trigger may or may not be identified. Conversely, the acute cardiac symptoms of secondary Takotsubo syndrome result from a complication of another primary condition or treatment. Secondary TTS commonly occurs in patients that have already been hospitalized for another ailment [1].

There are no universally accepted criteria for TTS, but several diagnostic criteria have been proposed. Takotsubo syndrome should be suspected in postmenopausal woman with the following symptoms: a clearly identified precipitant physical or emotional stress, an echocardiographic left ventricle dysfunction that is more severe than electrocardiogram (ECG) changes and the degree of cardiac biomarker elevation would indicate, acute cardiac chest pain with ST-segment elevation, and presentation of symptoms similar to acute coronary syndrome [5]. Anatomically, apical ballooning is the classic presentation as well as the most common. Variant forms of TTS such as midventricular, basal, and focal types have also been reported [6-7].

## Case presentation

A 33-year-old male with no significant medical history visited the emergency department (ED) presenting with weakness in the right side limbs, slurred speech, and transient loss of vision in his right eye. The patient reported it had occurred suddenly while at work, 30 minutes prior. He also complained of several episodes of bloody emesis and shortness of breath. The patient had a similar occurrence one week prior with only dizziness at the time. He works as a bartender and admitted to being under stress due to his large workload at the time of the incident. The patient drinks 2-3 drinks daily. He had smoked marijuana for four years. His average consumption was half of a joint daily, but his consumption increased the week prior to admission. On the day of the incident, he smoked two-and-a-half joints. 

**Figure 1:**
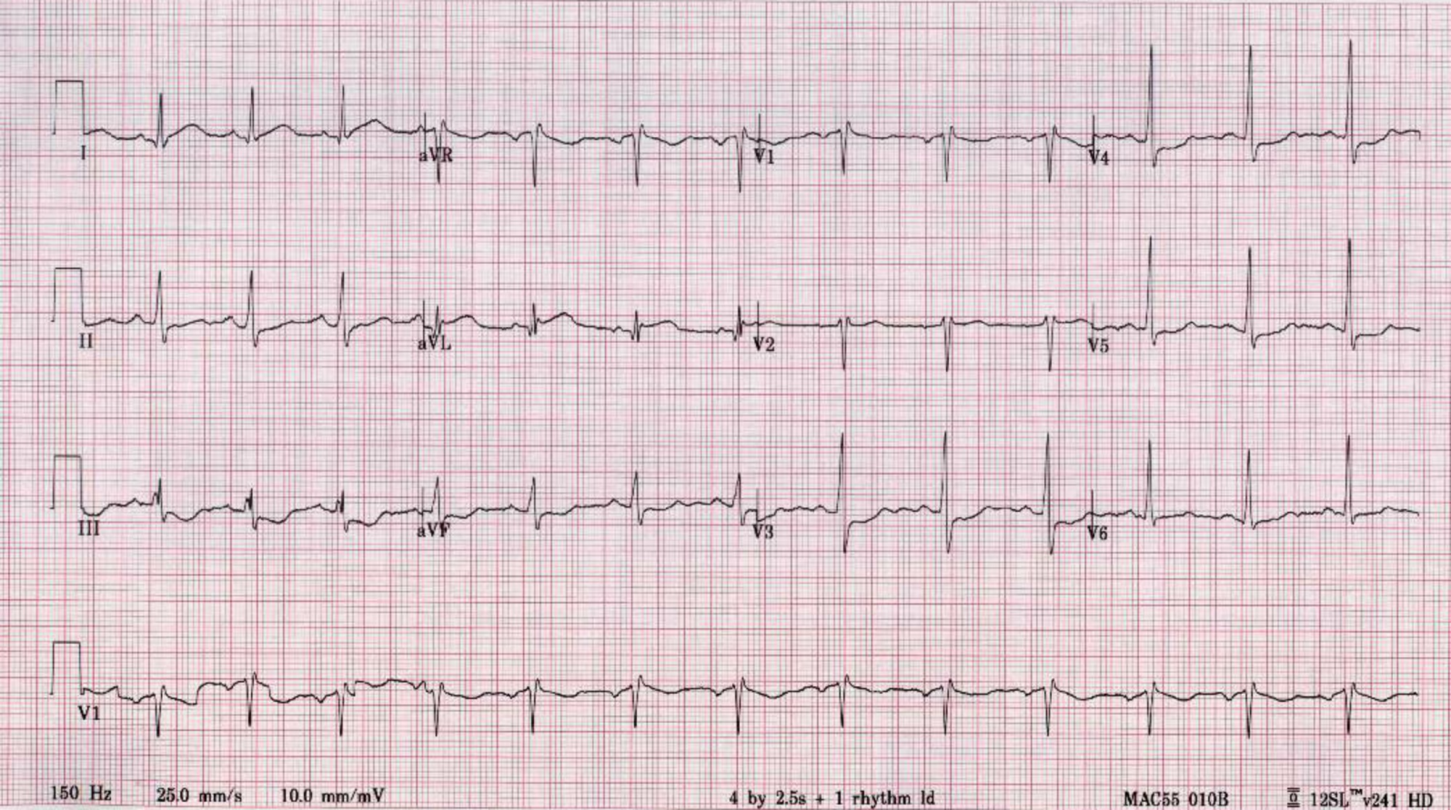
Electrocardiogram Acute ST-elevation myocardial infarction (STEMI)

**Figure 2:**
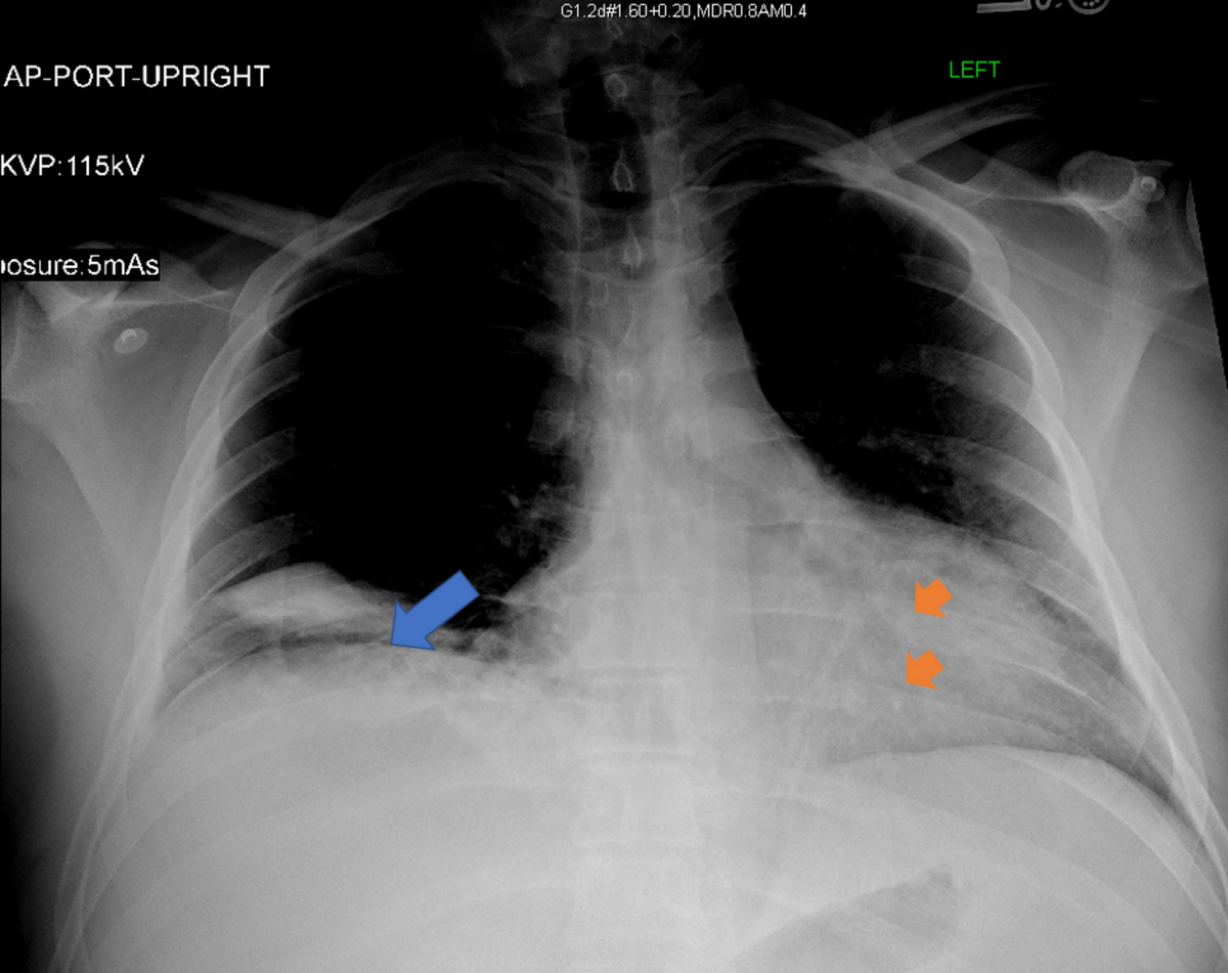
Chest X-ray Blue arrow pointing the pneumonic consolidation and orange arrows pointing some atelectasis

**Figure 3:**
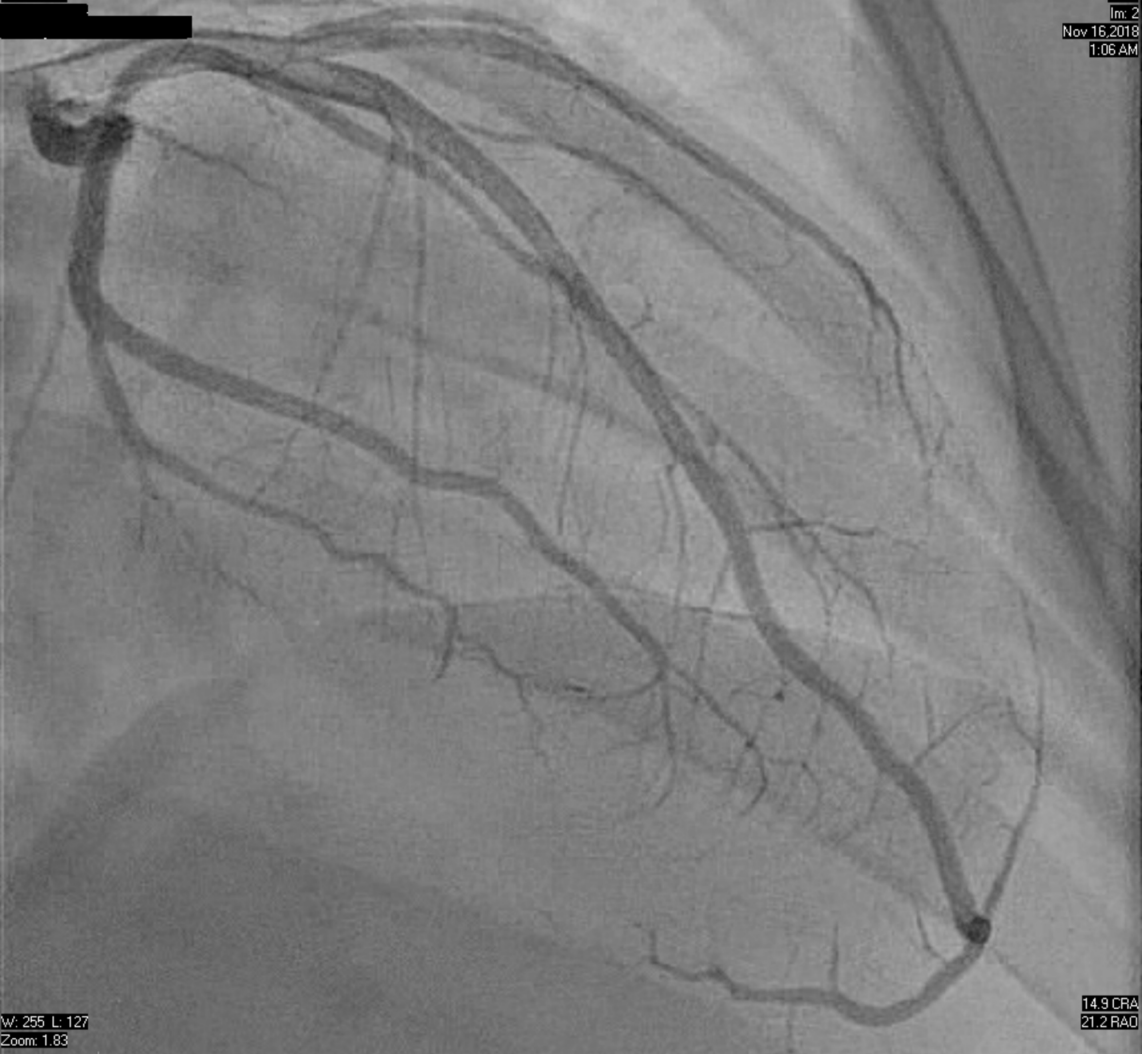
Coronary angiogram Normal coronary arteries

The electrocardiogram showed ST elevations in lead I and aVL, with depressions in the chest leads causing concern for possible lateral wall myocardial infarction (Figure 1). The patient presented with leukocytosis, hypokalemia, and elevated troponin levels. While in the ED, there was a discrepancy in arm blood pressure and a concern that an aortic dissection had formed. His chest X-ray showed no widening of the mediastinum, and confluent airspace opacity in the right lung base was suggestive of pneumonia. Additionally, patchy opacities located in the left lower lung were indicative of atelectasis (Figure 2). The patient’s CT scan showed patchy opacities which were suspicious for multifocal pneumonia, as well as a right artery dissection with multifocal stenosis and prominent bilateral neck lymph nodes. The patient became dyspneic and required oxygen. His neurological symptoms continued to wax and wane. A new electrocardiogram showed ST depression in leads II, III, avF, V3-V4, and ST elevation in V2. Troponin levels rose from 0.12 to 1.99 to 4.2. The patient was taken immediately to the catheterization laboratory, which found that the patient had normal coronaries. Mild left ventricular anteroapical akinesia was found through stress cardiomyopathy (Figure 3).

**Figure 4:**
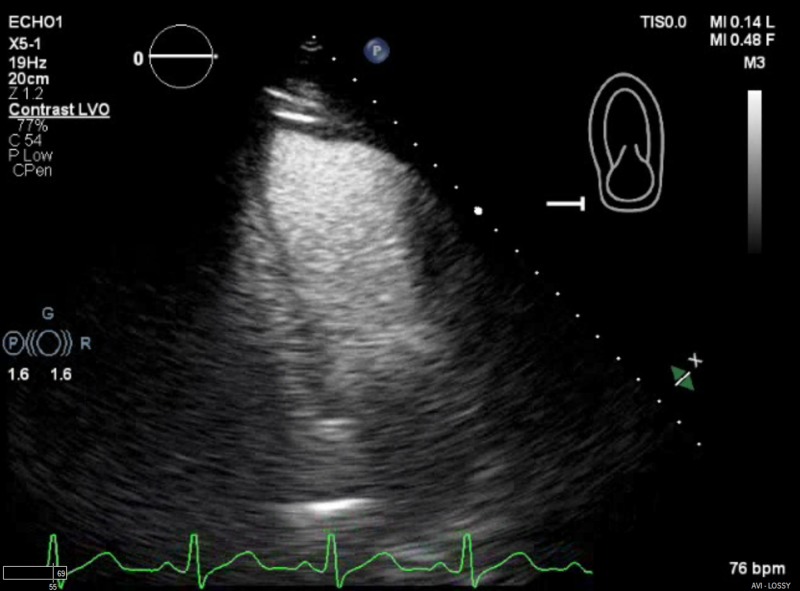
Echocardiogram Severe anterolateral and basal hypokinesia

The treatment was started with 2.5 mg of Lisinopril and 3.125 mg of carvedilol twice daily. The treatment was empiric due to a consolidation seen on X-ray. Ceftriaxone and Doxycycline were administered for possible community-acquired pneumonia. Compazine was given for nausea and vomiting. The patient’s echocardiography revealed a decreased left ventricular ejection fraction (LVEF 40%-45%) and severe anterolateral and basal hypokinesia (Figure 4). A cranial MRI discovered acute infarctions in the left lower ventral pons, the right middle cerebellar peduncle, and the middle and lower right cerebellar hemisphere. The neurology team recommended the use of anticoagulants for six months, to be bridged with Lovenox. The autoimmune workup was unremarkable. The Infectious Disease team was consulted for the incidental findings of multifocal pneumonia and a positive test for rhinovirus. Since the patient was asymptomatic, no further investigation or treatment was recommended. The patient was discharged on enoxaparin to bridge with 7.5 mg of Coumadin and 50 mg of metoprolol succinate to be taken daily. He was advised to avoid strenuous activity for at least six months and to have a follow-up neurology appointment in three months.

## Discussion

In the case presented, the patient´s Tokatsubo syndrome was triggered by emotional stressors. The patient met the InterTAK Diagnostic Score with 43 points (emotional trigger, physical trigger, and QTc prolongation) [8]. There are currently no clinical trials on the specific treatment of TTS. Current treatment strategies during the acute phase are mainly supportive, aiming to reduce life-threatening complications. According to the Heart Failure Association of the European Society of Cardiology, a high-risk patient is defined as an individual that has either one or more major risk factors or two or more minor risk factors. The major risk factors include an age of 75 years or older, a systolic blood pressure lower than 110 mm Hg, a clinical pulmonary edema, an LVEF of less than 35%; unexplained syncope, ventricular tachycardia, or ventricular fibrillation; left ventricular outflow tract obstruction ≥ 40 mm Hg, moderate or severe mitral regurgitation, apical thrombus, and new ventricular septal defect or contained left ventricular wall rupture. The minor risk factors include age between 70 and 75 years, LVEF of 35%-45%, preceding physical stressor, bystander obstructive coronary artery disease, biventricular involvement, electrocardiographic parameters (QTc ≥ 500 milliseconds, pathological Q waves and ST elevation for three or more days) and high levels of natriuretic peptides (BNP ≥ 600 pg/mL and NT-proBNP ≥ 2,000 pg/mL) [1]. Fortunately, this patient was not at high risk.

The associated risk factors for the patient are the age of greater than 55 years, smoking, alcohol abuse, anxiety states, and hyperlipidemia [9]. Our patient may have developed TTS secondary to heightened stress, high cannabis consumption, and vertebral dissection. Active marijuana use has been associated with a two-fold increased risk of developing TST. Despite being younger and having a more favorable cardiovascular risk profile, marijuana smokers are more likely to go into cardiac arrest or to be admitted into an Intensive Care Department than are nonusers [10].

Spontaneous coronary artery dissection (SCAD) is a differential diagnosis of TTS. Both can cause myocardial infarction with subsequent normalization of wall motion. However, the wall motion abnormality resulting from SCAD corresponds to the affected artery and may not have the classic apical ballooning appearance of TTS. In this case, we reviewed the images for an angiographic SCAD appearance and confirmed TTS diagnosis [11-12].

A cervical artery dissection is an intimal tear in a cervical artery (carotid or vertebral artery) with the development of an intramural hematoma that results in stenosis, occlusion, or aneurysmal dilation. Cervical artery dissection is an important cause of ischemic stroke in young and middle-aged adults. It is usually the result of neck trauma, frequently minor enough that the patient cannot recall it. Dissection of the vertebral artery (DVD) may present with pain in the posterior neck, headache, or ischemia in the distribution of the posterior circulation (vertigo, ataxia, swallowing difficulty). The diagnosis of DVD requires a high index of suspicion and may be confirmed with angiography, duplex scan, magnetic resonance angiography (MRA), or a CT scan. Antithrombotic therapy is recommended for at least three to six months for patients with ischemic stroke or TIA due to extracranial arterial dissection [13]. Only one other case of TTS with bilateral vertebral artery dissection has been reported [14].

Serious cardiac complications during the acute phase occur in approximately 20% of patients with TTS. Predictors for a poor outcome include physical triggers, pneumonia, acute neurological or psychiatric diseases, high troponin levels, and a low ejection fraction on admission [15]. Our patient had the latter two factors. Most patients with TTS have a spontaneous recovery of normal cardiac function. Patients with an LVEF greater than 45% and no complications can be discharged early. Higher-risk patients should be monitored in a coronary care or high-dependency unit with continuous electrocardiographic monitoring. Heart failure medications, such as beta blockers and angiotensin-converting enzyme inhibitors, should be considered for the treatment of lower-risk patients with reduced LVEF, as well as in higher-risk patients [16]. Beta blockers are prescribed for patients with left ventricular outflow obstruction resulting in hypotension. Management should be guided by patient hemodynamics and may also include fluids and phenylephrine. Patients with TTS should be followed up three months after discharge regardless of severity [17].

## Conclusions

We presented a case of a young man with vertebral artery dissection and TTS associated with marijuana use. TTS is a disease triggered by physical or emotional stressors. It can be confused with acute myocardial infarction and must be distinguished from this for the appropriate treatment to be administered. Once TTS has been properly diagnosed, the prognosis is generally favorable.
